# Incidence and Risk Factors of Elevated Intraocular Pressure Following Vitrectomy Surgery in Rhegmatogenous Retinal Detachment in a Tertiary Hospital in Northern Malaysia

**DOI:** 10.7759/cureus.98968

**Published:** 2025-12-11

**Authors:** Chang Feng Chew, Hong Kee Ng

**Affiliations:** 1 Department of Ophthalmology, Hospital Raja Permaisuri Bainun, Ipoh, MYS

**Keywords:** glaucoma, intraocular pressure, pars plana vitrectomy, rhegmatogenous retinal detachment, risk factors

## Abstract

Purpose

The purpose of this study was to determine the incidence and factors associated with early postoperative elevated intraocular pressure (IOP) following pars plana vitrectomy (PPV) for rhegmatogenous retinal detachment (RRD) at a tertiary hospital.

Methods

A single-centre retrospective observational study included 294 adults (aged ≥18 years) who underwent PPV with intraocular gas tamponade for RRD at Hospital Raja Permaisuri Bainun Ipoh between 2017 and 2023. Patients with pre-existing glaucoma, ocular hypertension, who had previous vitreoretinal surgery or silicone oil tamponade were excluded. IOP was measured at two to six hours, one week, and one month postoperatively and defined as elevated when IOP > 20 mmHg. Demographic data and surgical parameters were collected and analysed using Pearson’s chi-square and multiple logistic regression analyses to identify independent potential risk factors.

Results

A total of 98 eyes (33.3%) developed elevated IOP within one month postoperatively, peaking at one week (51.0%). Patients who were younger (<50 years) (OR: 3.19, 95% CI: 1.71, 5.93, p < 0.001), Chinese ethnicity (OR: 5.56, 95% CI: 1.71, 18.02, p = 0.004), and pseudophakic/aphakic status (OR: 2.16, 95% CI: 1.02, 4.58, p = 0.044), and absence of proliferative vitreoretinopathy (PVR) (OR: 1.83, 95% CI: 1.07, 3.13, p = 0.028) had statistical significance in IOP elevation. Gas tamponade type, postoperative lens status, surgery type, vitrectomy gauge, surgeon experience, and concomitant scleral buckle use had no statistical difference.

Conclusion

Elevated IOP occurred in one-third of eyes following PPV for RRD. Potential risk factors included younger age, Chinese ethnicity, pseudophakic or aphakic status, and absence of PVR. Early recognition of these factors may help prevent unwanted optic nerve damage, thus preventing potential vision loss.

## Introduction

The introduction of pars plana vitrectomy (PPV) in 1971 was a significant milestone in ophthalmology [1]. PPV is now considered one of the most common procedures after cataract and refractive surgeries [2]. Over the past five decades, the development of smaller-gauge vitrectomy systems - including 23-gauge (23G), 25-gauge (25G), and 27-gauge (27G) - and improvements in surgical techniques have significantly improved outcomes for retinal conditions. These include rhegmatogenous retinal detachment (RRD), macular holes (MH), and vitreous haemorrhage (VH) [1,3-7]. Despite these advancements, postoperative complications remain a concern. Among them, elevated intraocular pressure (IOP) is prevalent and clinically significant [1-6,8-12]. Postoperative IOP elevation is clinically significant because sustained or repeated IOP spikes may compromise optic nerve health and increase the long-term risk of secondary glaucoma, thus affecting vision.

The reported incidence of elevated IOP after PPV varies between 20% and 60% [2]. A 2017 systematic review found that 19-28% of patients developed ocular hypertension after vitreoretinal (VR) surgery [4]. Various studies have implicated factors, such as intraocular tamponade agents, inflammation, and steroid response; however, the incidence and risk factors for raised IOP following PPV remain incompletely defined, especially in cases limited to RRD [5,8,9]. Some recent studies have confirmed a higher incidence of ocular hypertension or open-angle glaucoma (OAG) after vitrectomy, whereas others have reported no increased long-term risk. Study cohorts differ: some select idiopathic epiretinal membrane (ERM) cases with or without an MH for homogeneity, while others include cases with diverse indications for vitrectomy, leading to inconsistent findings [1]. Overall, the current literature suggests that postoperative IOP elevation is common but variably reported, with uncertainty remaining specifically for RRD populations. This inconsistency underscores the importance of investigating postoperative IOP elevation specifically in RRD patients.

Postoperative IOP elevation following PPV may occur from both early and late mechanisms. Early postoperative IOP elevations are commonly related to factors such as gas expansion, retained viscoelastic material, inflammation, or transient pupillary block [2,8,9]. Late onset IOP elevation mechanisms include oxidative stress, trabecular meshwork dysfunction, and cytokine-mediated changes that may reduce aqueous outflow over time [6,13-16]. However, the relative contributions of these mechanisms remain uncertain, particularly in RRD populations, where published findings remain inconsistent.

Despite the growing number of vitrectomy procedures, few studies have specifically examined the incidence and risk factors for elevated IOP following PPV for RRD. This is particularly relevant in RRD, where gas tamponade is routinely used, and postoperative inflammation may be more pronounced, potentially increasing susceptibility to early postoperative IOP elevation. Furthermore, to the best of our knowledge, no study has explored this association in a multi-ethnic Southeast Asian population, such as Malaysia. Understanding the post-vitrectomy IOP response is crucial for identifying high-risk patients and implementing timely interventions. We therefore hypothesised that certain demographic and clinical characteristics, such as age, ethnicity, lens status, and intraoperative factors, would be associated with early postoperative IOP elevation in patients undergoing PPV for RRD. This study aimed to determine the incidence and factors associated with early postoperative elevated IOP following PPV for RRD in a tertiary hospital in Northern Malaysia, thereby addressing a key knowledge gap and informing local clinical practice.

## Materials and methods

This retrospective observational study was conducted at the Ophthalmology Department of Hospital Raja Permaisuri Bainun, Ipoh, Perak. We retrospectively retrieved data from the electronic surgical census and both electronic and physical medical records from January 2017 to December 2023. The local Ethics Committee of the National Medical Research Registry (NMRR) approved this study (registration number 24-01195-PTT and ID number 24-01505-A34). We conducted this study in accordance with the Declaration of Helsinki and Malaysian Good Clinical Practice Guidelines. Informed consent was waived by the ethics committee due to the retrospective nature of this study.

Sample size estimation was performed using the Scalex SP calculator by Naing et al. [17], based on an expected prevalence of 24% from Pillai et al. [5]. Using a 95% confidence level and 5% absolute precision, the minimum required sample size was 281 eyes. To account for an anticipated 10% loss, the target sample size was increased to approximately 313 eyes. The required minimum sample size was achieved in this study.

We included all patients aged ≥ 18 years who underwent PPV with intraocular gas tamponade for RRD between January 1, 2017, and December 31, 2023. These cases were recorded in the surgical electronic census. We excluded patients with a history of VR surgery, pre-existing ocular hypertension or glaucoma, those on ocular hypertensive medications, those with preoperative IOP > 20 mmHg, a follow-up period of less than one month, or who received intraocular tamponades aside from gas, such as air, balanced salt solution (BSS), or silicone oil (SO). We also excluded patients who required repeated VR surgery within one month of the initial procedure, or those with tractional or traumatic-induced RRD. Preoperative lens status was not an exclusion criterion.

We defined elevated IOP as IOP > 20 mmHg based on previous studies by Jamal et al. [4] and Pillai et al. [5] that used the same threshold to identify clinically meaningful postoperative IOP elevation and measured it using a Goldmann applanation tonometer [4,5]. We documented IOP at four time points: preoperatively, two to six hours, one week, and one month postoperatively, as these were routine postoperative assessments at our centre. We entered data into Microsoft Excel 2016 (Microsoft Corp., Redmond, WA) and analysed it using Statistical Package for Social Sciences (SPSS) version 26.0 (IBM Corp., Armonk, NY). We assessed the cumulative incidence of postoperative elevated IOP stepwise, removing eyes with previously elevated IOP from subsequent calculations. The overall cumulative one-month incidence represented the proportion of eyes that developed elevated IOP within the first postoperative month.

The primary outcome measured was the incidence of elevated IOP, defined as > 20 mmHg by Goldmann Applanation Tonometry postoperatively at two to six hours, one week, and one month postoperatively. We identified secondary outcomes as risk factors associated with elevated IOP.

We collected the following data: age, gender, ethnicity, type of intraocular gas tamponade, gauge of vitrectomy instrument, lens status at the end of vitrectomy surgery, surgeon experience, type of surgery (PPV ± cataract extraction), whether a concomitant scleral buckle was used, presence of proliferative vitreo-retinopathy (PVR), and IOP measurement preoperatively, at two to six hours, one week, and one month postoperatively. Surgeon experience was categorized based on training status: a consultant refers to a fully credentialed VR surgeon who has completed subspecialty training, whereas a fellow refers to a VR trainee still undergoing supervised subspecialty training.

We presented demographic and clinical profiles using descriptive statistics, including means ± standard deviations. We used the frequency method to determine the incidence and percentage of categorical data. We used Pearson’s chi-square test to compare categorical variables. We derived crude odds ratios (OR) and 95% CI from 2 × 2 contingency tables using SPSS Crosstabs (Risk Estimate) and simple logistic regression. All variables with clinical relevance or p < 0.20 in simple logistic regression analysis were included in the multiple logistic regression model. Multicollinearity was assessed using variance inflation factors (VIFs), and no significant multicollinearity was detected. The final multivariable model included 10 predictors with 98 outcome events, providing an events-per-variable ratio of approximately 10, which meets commonly accepted criteria for logistic regression model stability. We analysed the possible risk factors associated with elevated IOP using multiple logistic regression to identify potential independent risk factors. We performed all statistical analyses using SPSS version 26.0, and a value of p ≤ 0.05 was considered statistically significant.

## Results

A total of 320 eyes underwent PPV for RRD between January 1, 2017, and December 31, 2023, but only 294 eyes fulfilled the inclusion criteria. A total of 26 eyes were excluded due to pre-existing glaucoma or anti-glaucoma therapy, previous vitrectomy, early re-operation for redetachment, defaulted follow-ups, or incomplete IOP entries. The baseline demographic and clinical characteristics of the study population are summarized in Table 1. The mean age of patients without elevated IOP was slightly higher (56.9 ± 12.1 years) than that of patients with elevated IOP (53.9 ± 14.6 years). When stratified by age group, nearly half of our patients aged <50 years (49.3%) developed elevated IOP, compared with only 27.9% among those aged ≥50 years (p < 0.001).

**Table 1:** Demographics and basic characteristics of the study population Note: *p-value by Pearson’s chi-square test. Abbreviations: 23G, 23-gauge; 25G, 25-gauge; C3F8, perfluoropropane; IOP, intraocular pressure; PPV, pars plana vitrectomy; PVR, proliferative vitreoretinopathy; SD, standard deviation; SF6, sulfur hexafluoride

	Eyes without elevated IOP (%)	Eyes with elevated IOP (%)	p-value
	N = 196 (66.7%)	N = 98 (33.3%)	
Age (years; mean ± SD)	56.9 ± 12.1	53.9 ± 14.6	<0.001
<50	38 (50.7%)	37 (49.3%)	-
≥50	158 (72.1%)	61 (27.9%)	-
Gender			0.023
Male	107 (61.5%)	67 (38.5%)	-
Female	89 (74.2%)	31 (25.8%)	-
Ethnicity			<0.001
Malay	112 (71.8%)	44 (28.2%)	-
Chinese	59 (54.1%)	50 (45.9%)	-
Indian	25 (86.2%)	4 (13.8%)	-
Lens status			0.296
Phakic	53 (71.6%)	21 (28.4%)	-
Pseudophakic/aphakic	143 (65.0%)	77 (35.0%)	-
Tamponade			0.890
C3F8	177 (66.8%)	88 (33.2%)	-
SF6	19 (65.5%)	10 (34.5%)	-
PPV gauge			0.561
23G	107 (65.2%)	57 (34.8%)	-
25G	89 (68.5%)	41 (31.5%)	-
Surgeon experience			0.336
Consultant	172 (67.7%)	82 (32.3%)	-
Fellow	24 (60.0%)	16 (40.0%)	-
Surgery			0.319
PPV alone	106 (64.2%)	59 (35.8%)	-
PPV + cataract	90 (69.8%)	39 (30.2%)	-
PVR			0.005
Yes	116 (73.9%)	41 (26.1%)	-
No	80 (58.4%)	57 (41.6%)	-
Sclera buckle			0.097
Yes	48 (59.3%)	33 (40.7%)	-
No	148 (69.5%)	65 (30.5%)	-

Gender was significantly associated with postoperative IOP elevation, with 67 eyes (38.5%) in male patients demonstrating a higher incidence of elevated IOP than the 31 eyes (25.8%) in female patients (p = 0.023). Ethnicity also showed a statistically significant association with IOP elevation, with Chinese patients having the highest incidence of elevated IOP (45.9%), followed by Malay patients (28.2%), and Indian patients had the lowest incidence (13.8%; p < 0.001). This cohort consisted of 156 Malay (53.1%), 109 Chinese (37.1%), and 29 Indian (9.9%) patients.

Lens status postoperatively did not show a significant difference in IOP elevation between phakic (28.4%) and pseudophakic/aphakic (35.0%) patients (p = 0.296). Similarly, the type of intraocular gas tamponade used was not statistically significant, with 33.2% in the perfluoropropane (C3F8) gas group and 34.5% in the sulfur hexafluoride (SF6) gas group (p = 0.890).

Elevated IOP was observed in 34.8% of 164 eyes that underwent PPV with a 23G vitrectomy, compared with 31.5% of 130 eyes with the 25G system; this difference was not statistically significant (p = 0.561). Surgeon experience was also not statistically significant for IOP elevation, with 32.3% and 40% of the consultant and fellow groups, respectively, experiencing elevated IOP (p = 0.336). Increased IOP was observed in 30.2% of eyes that underwent combined PPV with cataract surgery, compared with 35.8% of those who received PPV alone; however, this difference was not statistically significant (p = 0.319).

Patients without intraoperative proliferative vitreoretinopathy (PVR) had a higher incidence (41.6%) than those with PVR (26.1%; p = 0.005). Although patients who received concomitant scleral buckle had a higher incidence of elevated IOP (40.7%) than those without (30.5%), this difference did not reach statistical significance (p = 0.097).

Table 2 summarizes the chi-square analyses for categorical variables. Significant associations were observed for age group, gender, ethnicity, and presence of PVR (p < 0.05). Degrees of freedom and effect sizes are reported for each comparison.

**Table 2:** Pearson chi-square analysis of factors associated with postoperative IOP Note: χ², Pearson’s chi-square; Φ, Phi coefficient for 2 × 2 tables; V, Cramer’s V for tables > 2 × 2 Abbreviations: df, degree of freedom; IOP, intraocular pressure; PPV, pars plana vitrectomy; PVR, proliferative vitreoretinopathy

Variable	χ²	df	p-value	Effect size (Φ/V)
Age	11.60	1	0.001	0.199
Gender	5.13	1	0.023	0.132
Ethnicity	14.54	2	0.001	0.222
Lens status	1.09	1	0.296	0.061
Gas type	0.02	1	0.890	0.008
PPV gauge	0.34	1	0.561	0.034
Surgeon experience	0.93	1	0.336	0.056
Surgery type (PPV vs PPV + cataract)	1.00	1	0.319	0.058
PVR status	7.90	1	0.005	0.164
Scleral buckle	2.76	1	0.097	0.097

Incidence of elevated IOP > 20 mmHg

In the one-month postoperative period, 98 eyes (33.3%) had elevated IOP. It was observed that 23 of 294 eyes (7.8%) had elevated IOP at two to six hours, 50 of 271 eyes (18.5%) at one week, and 25 of 221 eyes (11.3%) at one month postoperatively. The cumulative incidence rates are summarized in Table 3. The peak IOP elevation occurred at one week post-PPV in 50 of 98 eyes (51.0%). 

**Table 3:** Cumulative incidence rate and overall incidence of elevated IOP post-PPV Abbreviations: IOP, intraocular pressure; PPV, pars plana vitrectomy

Postoperatively IOP measurement	New cases with elevated IOP (N)	Eyes at risk (N)	Incidence (%)
Two to six hours	23	294	7.8
One week	50	271	18.5
One month	25	221	11.3
Overall one month	98	294	33.3

Possible risk factors for IOP elevation post-PPV

Pearson’s chi-square testing identified several factors significantly associated with elevated IOP after PPV for RRD. The possible associated factors are summarized in Table 4. Crude OR and 95% CI were derived from 2 × 2 contingency tables using SPSS Crosstabs (Risk Estimate). For ethnicity (a three-category variable), crude ORs were obtained using simple logistic regression. A multiple logistic regression was then performed to identify potential independent risk factors, which are also summarized in Table 4.

**Table 4:** Risk factors of elevated IOP post-PPV in RRD patients Note: Crude odds ratios (ORs) and 95 % confidence intervals (CIs) were calculated from 2 × 2 contingency tables using the Risk Estimate function in SPSS Crosstabs for binary variables. Adjusted ORs and 95% CI were determined using multiple logistic regression analysis. Lens status refers to whether the patient was phakic, pseudophakic, or aphakic after PPV. *Crude ORs and 95 % CIs were obtained using univariate binary logistic regression for ethnicity (a three-category variable). Abbreviations: 23G, 23-gauge; 25G, 25-gauge; C3F8, perfluoropropane; CI, confidence interval; IOP, intraocular pressure; OR, odds ratio; PPV, pars plana vitrectomy; PVR, proliferative vitreoretinopathy; SF6, sulfur hexafluoride

Risk factors	Frequency, N (%)		Crude OR (95% CI)	p-value	Adjusted OR (95% CI)	p-value
	Normal IOP	Elevated IOP				
Age						
<50	38 (50.7%)	37 (49.3%)	2.52 (1.47, 4.33)	<0.001	3.19 (1.71, 5.93)	<0.001
≥50	158 (72.1%)	61 (27.9%)	(reference)	-	(reference)	-
Gender						
Male	107 (61.5%)	67 (38.5%)	1.80 (1.08, 2.99)	0.023	1.71 (0.98, 2.98)	0.058
Female	89 (74.2%)	31 (25.8%)	(reference)	-	(reference)	-
Ethnicity*						
Malay	112 (71.8%)	44 (28.2%)	(reference)	-	(reference)	-
Chinese	59 (54.1%)	50 (45.9%)	2.16 (1.28, 3.61)	0.004	5.56 (1.71, 18.02)	0.004
Indian	25 (86.2%)	4 (13.8%)	0.41 (0.13, 1.24)	0.113	2.44 (0.76, 7.84)	0.134
Tamponade agents						
C3F8	177 (66.8%)	88 (33.2%)	0.95 (0.42, 2.12)	0.890	0.787 (0.32, 1.92)	0.600
SF6	19 (65.5%)	10 (34.5%)	(reference)	-	(reference)	-
PPV gauge						
23G	107 (65.2%)	57 (34.8%)	(reference)	-	(reference)	-
25G	89 (68.5%)	41 (31.5%)	0.87 (0.53, 1.41)	0.561	1.00 (0.58, 1.71)	0.992
Lens status						
Phakic	53 (71.6%)	21 (28.4%)	(reference)	-	(reference)	-
Pseudophakic/aphakic	143 (65.0%)	77 (35.0%)	1.36 (0.76, 2.42)	0.296	2.16 (1.02, 4.58)	0.044
Surgeon Experience						
Consultant	172 (67.7%)	82 (32.3%)	0.72 (0.36, 1.42)	0.336	0.80 (0.37, 1.73)	0.567
Fellow	24 (60.0%)	16 (40.0%)	(reference)	-	(reference)	-
Surgery						
PPV alone	106 (64.2%)	59 (35.8%)	1.28 (0.79, 2.10)	0.319	1.81 (0.96, 3.41)	0.068
PPV + cataract	90 (69.8%)	39 (30.2%)	(reference)	-	(reference)	-
PVR						
Yes	116 (73.9%)	41 (26.1%)	(reference)	-	(reference)	-
No	80 (58.4%)	57 (41.6%)	2.02 (1.23, 3.30)	0.005	1.83 (1.07, 3.13)	0.028
Sclera Buckle						
Yes	48 (59.3%)	33 (40.7%)	1.57 (0.92, 2.66)	0.097	1.09 (0.59, 2.01)	0.790
No	148 (69.5%)	65 (30.5%)	(reference)	-	(reference)	-

Younger patients (age < 50 years) were significantly more likely to develop raised IOP post-PPV compared to older patients, with a value of p < 0.001 and an OR of 2.52 with a 95% CI (1.47, 4.33). Male patients had a higher likelihood of developing elevated IOP than females (p = 0.023), OR 1.80; 95% CI (1.08, 2.99). Among the multi-ethnicities in our cohort, Malay was set as the reference, and Chinese were statistically significant (p = 0.004), with approximately two times the likelihood to develop high IOP postoperatively, OR 2.156; 95% CI (1.28, 3.61). Indians, however, were not statistically significant (p = 0.113), OR 0.41; 95% CI (0.13, 1.24). Patients without PVR were two times more likely to develop elevated IOP following PPV (p = 0.005) with an OR 2.02 and a 95% CI (1.23, 3.30).

Other parameters were not statistically significant: tamponade agents (C3F8 versus SF6), PPV gauge (23G versus 25G), lens status postoperatively (phakic versus pseudophakic or aphakic), surgeon’s experience (consultant versus fellow), type of surgery (vitrectomy alone versus combined cataract and vitrectomy), and patients who received scleral buckle versus those who did not. These are summarized in Table 4.

Multiple logistic regression was performed to identify potential independent risk factors, along with adjusted ORs and 95% CIs. Younger age, Chinese ethnicity, postoperative pseudophakia or aphakia, and the absence of PVR during surgery were significantly associated with elevated IOP post-PPV. Patients younger than 50 years were 3 times more at risk of developing raised IOP postoperatively than older patients (p < 0.001), OR 3.19; 95% CI (1.71, 5.93). Our male cohorts were 1.7 times more likely to experience elevated IOP than the females, but this difference was not statistically significant after multiple logistic regression analysis (p = 0.058), OR 1.71; 95% CI (0.98, 2.98). In our multi-ethnic cohort, Chinese patients were 5.5 times more likely to experience elevated IOP (p = 0.004), OR 5.56; 95% CI (1.71, 18.02), with Malay ethnicity as the reference. Following multiple logistic regression, patients who were either pseudophakic or aphakic post-PPV were significantly associated with raised IOP (p = 0.044), OR 2.16; 95% CI (1.02, 4.58). Patients without PVR intraoperatively were also a potential risk factor for developing elevated IOP postoperatively (p = 0.028), OR 1.83; 95% CI (1.07, 3.13). The other parameters mentioned earlier remained statistically insignificant after running multiple logistic regression analyses, as summarized in Table 4.

## Discussion

Elevated IOP following VR surgery is a well-recognized complication; however, existing literature presents conflicting evidence regarding the relationship between vitrectomy and postoperative IOP elevation [5,8,13]. In our retrospective study, the incidence of elevated IOP following PPV for RRD was 33.3% at one month postoperatively, which corresponds with earlier studies, where post-vitrectomy IOP elevation ranged between 20% and 60% depending on case selection and definitions used [1-6]. Pillai et al. [5] reported an incidence of 24.25% in an Indian cohort with mixed indications for PPV, while another study [1] reported a 19.2% rate in eyes with idiopathic ERM. Xu et al. [9] reported an incidence of 35% raised IOP within one week postoperatively, peaking at one day post-PPV (57.4%). Our study showed a similar outcome, with a peak of 51.0% of the 98 eyes developing elevated IOP at one week postoperatively. Our higher incidence likely reflects both the acute nature of RRD surgery and the predominance of intraocular gas tamponade, which is a known cause of transient IOP rise [9].

Recent studies have suggested multiple mechanisms underlying post-vitrectomy IOP elevation. In the early postoperative period, studies have reported that postoperative inflammatory debris may occlude the trabecular meshwork, limiting aqueous outflow and leading to an increase in IOP [13,14,16]. Other causes include retained viscoelastic substances, gas expansion, haemorrhage, or steroid response [2,8,9]. Additionally, fibrin formation or transient pupillary block could contribute to early IOP spikes [2,8,9]. These mechanisms align with our finding that more than half of postoperative IOP elevations occurred within the first week.

In the late postoperative period, an increase in oxygen levels in the vitreous cavity following PPV has been reported, leading to oxidative stress and damage to the trabecular meshwork [14-16]. Concurrently, cytokines and chemokines are released as the damaged trabecular meshwork undergoes repair, resulting in fibrosis and stiffness of the trabecular meshwork, causing further mechanical stress [14,15]. This is further supported by Jamal et al. [4], where a systematic review in 2017 revealed evidence that PPV is a risk factor for ocular hypertension and OAG, with oxidative stress being the main instigator [4].

In our study, younger age was found to be a possible risk factor, with patients under 50 years showing a threefold increased likelihood of experiencing elevated IOP following PPV. This observation is consistent with previous studies demonstrating greater susceptibility to elevated IOP in younger patients [2,5]. Proposed explanations include stronger vitreous base adhesion and heightened inflammatory response, which may predispose younger eyes to postoperative trabeculitis [8,9].

Ethnicity was significantly associated with postoperative IOP elevation, with Chinese patients 5.5 times more likely to develop elevated IOP compared to our Malay cohort, while Indians had the lowest incidence. To the best of our knowledge, this ethnic variability has not been widely reported in the literature, making this a novel finding of the present study. Previous studies have shown that Chinese ethnicity is a risk factor for angle closure, likely due to a smaller anterior chamber depth and shorter axial length [18,19]. This predisposes Chinese patients to IOP spikes due to angle closure when intraocular gas tamponade is performed. The observed association between Chinese ethnicity and elevated IOP may reflect unmeasured anatomical factors or surgical variables. Given our retrospective design, causality cannot be inferred. This finding should therefore be interpreted cautiously and validated in future prospective studies.

Pseudophakic or aphakic lens status was twice as likely to develop elevated IOP compared to phakic eyes. This is consistent with prior reports that pseudophakia increases trabecular meshwork exposure to oxidative stress and inflammatory changes after vitrectomy [4,6,9]. Weinreb et al. [20] and Siegfried et al. [21] further demonstrated that oxidative stress and low-grade inflammation contribute to trabecular dysfunction, with pseudophakic eyes being particularly susceptible, as the crystalline lens acts as an oxidative stress scavenger [6,13,14].

Interestingly, the absence of proliferative vitreoretinopathy (PVR) was associated with an increased risk of IOP elevation, which is another novel finding of this study. The inverse association between PVR and elevated IOP is likely influenced by selection bias. In our center, eyes with more advanced or recurrent RRD, often associated with PVR, are more commonly managed with silicone oil rather than gas tamponade, and all silicone oil cases were excluded from this cohort. Silicone oil tamponade has been repeatedly identified as a risk factor for secondary glaucoma after VR surgery and is typically reserved for more complex RRDs [2,5,8,9,12]. In addition, eyes requiring early repeat VR surgery within one month, again more likely to represent severe PVR or recurrent detachment, were also excluded. Therefore, this finding should be interpreted with caution. Other unmeasured confounders, including the extent of intraoperative laser or cryotherapy and the degree of inflammation, may contribute to postoperative IOP elevation. This warrants further investigation. Our study findings did not align with a previous study where PVR in RRD was reported as a risk factor for IOP elevation [9]. Two other recent studies have described the role of cytokines and chemokines in PVR in relation to high IOP after PPV for RRD. Interleukin-6 (IL-6) has been identified as a potential key player and is significantly associated with high IOP post-PPV in RRD [14,16].

Although gender showed an association in the earlier chi-square analysis, which corresponds with Pillai et al. [5], this did not persist after adjustment, suggesting that the initial finding may have been confounded by other factors. Our findings did not show significant differences between 23G and 25G vitrectomy, which aligns with Kovacic et al. [10]. It was postulated that the smaller sclerotomy wounds are not sutured; therefore, they may result in more wound leakage, causing transient hypotony until the wound heals spontaneously. Kovacic et al. [10] also described no significant difference between types of gas tamponade, just as in our study. This could be due to the much smaller sample size in the SF6 subgroup. Another possibility is due to the non-expansile gas concentration used in our centre (C3F8: 12-16% and SF6: 20-24%).

In terms of surgeon experience, we reported that fellows had a slightly higher incidence of IOP elevation than consultants (40.0% and 32.3%, respectively), but this difference was not statistically significant. This could be due to our consultants handling more complex cases, which may require more manipulation and longer intraoperative time, or the fellows receiving proper supervision. To the best of our knowledge, no other studies have reported on the association between surgeons’ experience with PPV and elevated IOP. The use of a concomitant scleral buckle with PPV is common in managing RRD. In our study, we reported no significant association with elevated IOP, which aligns with earlier studies [5,9,11]. Conflicting results have also been reported, where scleral buckling was identified as a risk factor, where it was mentioned that the vortex vein was constricted, thus increasing the episcleral venous pressure [2,8,9,11,13]. This difference could be due to the variation in study design, case selection, and the experience of the surgeon.

Our findings align with previous studies, which have shown that IOP elevation following PPV is multifactorial, involving mechanisms such as gas expansion, trabecular meshwork obstruction, oxidative stress, and inflammatory changes [2,3,8,9,12]. The clinical significance lies in its potential progression to secondary glaucoma, as confirmed by long-term population-based studies linking PPV to a higher risk of glaucoma [6,11]. Our findings suggest that certain patient groups, i.e., younger patients of Chinese ethnicity who are pseudophakic or aphakic without PVR, may warrant careful postoperative observation within the first postoperative week. However, the role of tailored follow-up or prophylactic IOP-lowering therapy requires confirmation in future prospective studies.

Strengths and limitations

The strengths of our study include a relatively large sample size and a multi-ethnic Malaysian cohort focusing only on RRD with intraocular gas tamponade cohorts, which provides unique insights that have not been widely reported in previous studies. The limitations of this study include its retrospective design, which may introduce selection bias; reliance on available medical records, which may result in incomplete data; and the fact that it was conducted at a single tertiary centre, which may limit generalizability. Furthermore, only short-term (one-month) IOP outcomes were assessed, and therefore, the findings cannot be extrapolated to long-term glaucoma risk, whereas long-term studies suggest that chronic IOP elevation and subsequent glaucoma may develop years after vitrectomy [1,5,11,13]. The multiple logistic regression model includes several variables relative to the number of events and may be prone to overfitting. Another limitation is the wide variation in the definition of elevated IOP across studies, highlighting the need for a consensus, as this variability can affect research findings and comparisons [5]. Multiple surgeons may have introduced confounding factors through variable surgical techniques, potentially affecting our findings. The exclusion of silicone oil tamponade and patients undergoing early reoperation may introduce collider bias affecting PVR interpretation. We did not include diabetic retinopathy status, duration of surgery, or intraoperative procedures such as cryotherapy and endolaser, as well as anatomical parameters such as anterior chamber depth and axial length, as these may also influence the outcome [5,10,15]. Postoperative medications were also not taken into account, which could confound the outcome.

## Conclusions

Elevated IOP following VR surgery for RRD is a common and multifactorial complication. In this study, one-third of the patients experienced elevated IOP within one month. Younger age, Chinese ethnicity, pseudophakia or aphakia, and absence of PVR were associated with a higher likelihood of elevated IOP following PPV. These findings suggest that closer postoperative observation may be warranted in these groups to allow early detection and timely management, although whether this approach reduces the risk of vision-threatening outcomes requires further study. Future prospective multicentre studies with longer follow-up periods are needed to confirm these associations and evaluate the long-term risk of glaucoma before formal screening recommendations can be considered.
